# Risk of neurodegenerative disease or dementia in adults with attention-deficit/hyperactivity disorder: a systematic review

**DOI:** 10.3389/fpsyt.2023.1158546

**Published:** 2023-08-17

**Authors:** Sara Becker, Mohammad Chowdhury, Pattara Tavilsup, Dallas Seitz, Brandy L. Callahan

**Affiliations:** ^1^Department of Psychology, University of Calgary, Calgary, AB, Canada; ^2^Hotchkiss Brain Institute, University of Calgary, Calgary, AB, Canada; ^3^Department of Psychiatry, Cumming School of Medicine, University of Calgary, Calgary, AB, Canada

**Keywords:** ADHD, dementia, Lewy body dementia, Parkinson’s disease, Alzheimer’s disease, vascular dementia, older adults, mild cognitive impairment

## Abstract

**Purpose of review:**

Several psychiatric disorders have been associated with an increased risk of developing a neurodegenerative disease and/or dementia. Attention-deficit/hyperactivity disorder (ADHD), a neurodevelopmental disorder, has been understudied in relation to dementia risk. We summarized existing literature investigating the risk of incident neurodegenerative disease or dementia associated with ADHD.

**Recent findings:**

We searched five databases for cohort, case–control, and clinical trial studies investigating associations between ADHD and neurodegenerative diseases/dementia in May 2023. Study characteristics were extracted by two independent raters, and risk of bias was assessed using the Newcastle Ottawa Scale. Search terms yielded 2,137 articles, and seven studies (five cohort and two case–control studies) ultimately met inclusion criteria. Studies examined the following types of neurodegeneration: all-cause dementia, Alzheimer’s disease, Parkinson’s and Lewy body diseases, vascular dementia, and mild cognitive impairment. Heterogeneity in study methodology, particularly covariates used in analyses and types of ratios for risk reported, prevented a meta-analysis and data were therefore summarized as a narrative synthesis. The majority of studies (4/7) demonstrated an overall low risk of bias.

**Summary:**

The current literature on risk of developing a neurodegenerative disease in ADHD is limited. Although the studies identified present evidence for a link between ADHD and subsequent development of dementia, the magnitude of the direct effect of ADHD on neurodegeneration is yet to be determined and better empirically designed studies are first needed. Furthermore, the mechanism of how or why ADHD is associated with an increased risk of developing a neurocognitive disorder is still unclear and should be explored in future studies.

**Systematic review registration:**

https://www.crd.york.ac.uk/prospero/display_record.php?ID=CRD42022348976, the PROSPERO number is CRD42022348976.

## 1. Introduction

With the aging population, the global prevalence of neurodegenerative diseases and dementia is on the rise (1). Dementia is characterized by a progressive decline in cognitive function that presents a significant change from the person’s prior level of functioning that impairs their ability to function independently in society (i.e., impairs their activities of daily living function) (2). Cognitive changes are often most evident in the memory domain, but also affect attention, executive functions, visuo-perception, and language skills. There are many different types of neurodegenerative disorders and dementia, including Alzheimer’s disease (AD), vascular dementia (VaD), Lewy body diseases (LBD) encompassing dementia with Lewy bodies (DLB) and Parkinson’s disease (PD) with or without dementia, frontotemporal dementia, and more (2).

In 2019, it was estimated that 55 million people were living with dementia worldwide, a number that is expected to increase to 78 million by 2030 and 139 million by 2050 (3). This poses a huge burden not only on patients, but also on their families and caregivers who will suffer from increasing caregiver burden and burnout, leading to poor physical and psychological health as well as increased social isolation and financial difficulties (4, 5). Additionally, dementia is an enormous burden on the healthcare system; in 2018 the cost of dementia was estimated at US $1 trillion and is estimated to surpass US $2 trillion by 2030 (6, 7). The ability to identify those at risk for future dementia is therefore crucial for improving the lives of patients and families and alleviating the global social and economic burden of dementia.

Although age is the strongest risk factor for dementia (6, 8), dementia is not an inevitable consequence of aging. A landmark study by the Lancet Commission (9) highlighted 12 potentially modifiable risk factors that could delay or prevent the development of dementia by up to 40%. Some of the strongest risk factors identified included hearing loss [relative risk (RR) 1.9, 95% confidence interval (CI) 1.4–2.7], depression (RR 1.9, 95% CI 1.6–2.3), and traumatic brain injury (RR 1.8, 95% CI 1.5–2.2). While this study recognized depression as one of the 12 modifiable risk factors, other psychiatric disorders were not identified. However, emergent research has been increasingly showing a relationship between other psychiatric disorders, such as anxiety or bipolar disorder, and development of dementia (10). For instance, a diagnosis of bipolar disorder is associated with an almost 3-fold increase in risk of developing dementia [Odds Ratio (OR) 2.96, 95% CI 2.09–4.18] (11). A population-based cohort study in Denmark showed schizophrenia was associated with a more than 2-fold higher risk of all-cause dementia after adjusting for age, sex, and calendar period (IRR 2.13, 95% CI 2.00–2.27) (12). Furthermore, a recent meta-analysis found that participants with schizophrenia had significantly higher risk of developing dementia (combined RR 2.29, 95% CI 1.35–3.88), compared with participants who did not have schizophrenia (13). Anxiety predicted incident cognitive impairment (RR 1.77, 95% CI 1.38–2.26) (14) and dementia (RR 1.57, 95% CI 1.02–2.42) in one meta-analysis, and a more recent meta-analysis showed that the overall RR of dementia was 1.24 (95% CI 1.06–1.46) in participants with anxiety (15).

In the last decade, studies have emerged identifying attention-deficit/hyperactivity disorder (ADHD), one of the most common neurodevelopmental disorders, as a new psychiatric condition that additionally may increase the risk of later development of dementia (16, 17). ADHD, classically considered a disorder of childhood, is characterized by core symptoms of attention, impulsivity, and hyperactivity (18, 19). These symptoms persist into adulthood in about 40–60% of cases (20, 21) and even persist into later life, with around 3% of adults aged 50 and older reporting clinically significant ADHD symptoms (22, 23). We recently undertook a critical appraisal of studies showing the first associations between ADHD in adults and dementia risk (16); however, to the best of our knowledge, no study has systematically reviewed the overall risk of dementia in people with ADHD. To ascertain a comprehensive estimate of the influence of ADHD on dementia risk, we undertook a systematic review and meta-analysis of studies to quantify the risk of incident neurodegenerative disease or dementia associated with ADHD, relative to the general population.

## 2. Methods

This study was conducted and reported according to the Preferred Reporting Items for Systematic Reviews and Meta-Analyses (PRISMA) 2020 guidelines (24). The review protocol was previously published in PROSPERO,^1^ registration number CRD42022348976.

### 2.1. Search strategy

To identify relevant articles, we used key words and medical subject headlines (see Supplementary File 1) to search the following databases: MEDLINE, PsychINFO, Scopus, Web of Science, and Google Scholar. A broad range of databases was chosen to minimize selection bias, and these five were chosen because they are commonly used in the health sciences. Searches were limited to human studies. No restrictions were made regarding publication period or language of publication. A first search was conducted between August 23 and September 27, 2022, and an updated search was conducted May 17, 2023. All articles were imported into the Covidence software platform (Covidence systematic review software, Veritas Health Innovation, Melbourne, Australia) for screening and full text review.

Inclusion criteria were: (1) study design was either cohort study, clinical trial, or case–control study, (2) participants were adults (aged 18+) with ADHD diagnosed using standardized clinical criteria, and (3) outcome was a neurodegenerative disorder and/or dementia diagnosed using standardized clinical criteria. Studies were excluded if the study population was comprised only of people <18 years of age, or if the publication was a systematic review, meta-analysis, conference abstract, book, book chapter, editorial, case study, case series, opinion, or dissertation/thesis.

### 2.2. Definition of exposure and outcomes

Adults who received a clinical diagnosis of ADHD per standardized criteria, defined using the Diagnostic and Statistical Manual of Mental Disorders (DSM) (25) or the International Classification of Diseases (ICD) (26) coding system, were considered ‘exposed’ to ADHD. All versions of the DSM or the ICD were accepted. Diagnoses could have been made as an adult or a child. Adults without a diagnosis of ADHD were considered unexposed controls.

The primary outcome was the development of a neurodegenerative disorder or dementia [including, but not limited to, mild cognitive impairment (MCI), AD, PD with or without dementia, DLB, VaD, Frontotemporal lobar degeneration, etc.]. Outcomes were defined using relevant standardized clinical criteria: DSM or ICD codes, or validated consensus criteria [e.g., the National Institute on Aging criteria for AD (27), the DLB Consortium criteria for DLB (28), or the Neuroepidemiology Branch of the National Institute of Neurological Disorders and Stroke criteria for VaD (29)].

### 2.3. Study selection

Titles and abstracts of studies retrieved using the above search strategy were each screened by two of six raters who were blinded to others’ ratings, to ascertain inclusion criteria. All articles either not fulfilling all inclusion criteria, or fulfilling any of the exclusion criteria, were excluded. Manuscripts were assessed independently, and disagreements were resolved between the two screening raters. Potentially eligible studies were retrieved, and the full text was then further assessed for eligibility by two independent raters. All disagreements were resolved through discussion between the two raters.

### 2.4. Data extraction

Two review authors independently extracted data from retained studies into a standardized form for assessment of study quality and evidence synthesis. Any discrepancies were identified and resolved through discussion, arbitered by a third author where necessary. Extracted information included: aims, setting, population, methodology (including inclusion and exclusion criteria), recruitment and study completion rates where applicable, operationalized definitions of ADHD (exposure) and neurodegeneration and/or dementia (outcome), number of exposed (ADHD) and unexposed (control) participants as well as their characteristics (age, % female sex, education years, and race/ethnicity), total number of outcomes where applicable, odds/risk ratios – crude and adjusted – reported including variables that were controlled or covaried for, and related information for assessment of the risk of bias. Missing data regarding exposure and outcomes for three studies (30–32) were requested from study authors.

### 2.5. Risk of bias assessment

Each study included was independently assessed by two researchers using the Newcastle-Ottawa Quality Assessment Scale (NOS) for case–control and cohort studies (33). The NOS assesses quality of each study using a ‘star’ system that judges eight items categorized into three main aspects: (1) selection of study groups, (2) comparability of groups based on the design or analyses, and (3) ascertainment of either exposure for case–control studies, or outcome of interest for cohort studies. A maximum of nine ‘stars’ can be awarded, denoting the highest quality. Risk of bias was determined based on the amount of ‘stars’ awarded: high risk of bias 0–3, medium risk 4–6, and low risk 7–9. For (randomized) clinical trials, we planned to use the revised Cochrane risk-of-bias tool which assesses quality as a judgment (high, low, some concerns) for items in six separate domains (34).

### 2.6. Data synthesis and meta-analysis

We planned to synthesize the effect measures (e.g., odds ratios, risk ratios, incidence rate ratios) collected from the final selected studies through a meta-analysis. This would provide us with a pooled estimate (weighted average) of the effect measures (e.g., a weighted average of hazard ratios derived from multiple similar studies that presented the same hazard ratios), and we would be able to obtain an overall/summary estimate of the association between our exposure (ADHD) and outcome (neurodegenerative disorders/dementia). Furthermore, we wanted to specify in the meta-analysis if the odd ratios/risk ratios reported in the individual studies were crude or adjusted, and what factors were adjusted for in the individual studies.

### 2.7. Analysis of subgroups

We planned to undertake the following subgroup analyses to see if effect measures vary across these factors: by age, by gender, by ethnicity, by neurodegenerative disease subtypes (e.g., AD, PD, DLB, all-cause dementia), by study design (cross-sectional vs. case control vs. cohort), by study quality, and by diagnostic criteria applied (e.g., DSM, ICD).

## 3. Results

### 3.1. Study selection

**Figure 1:**
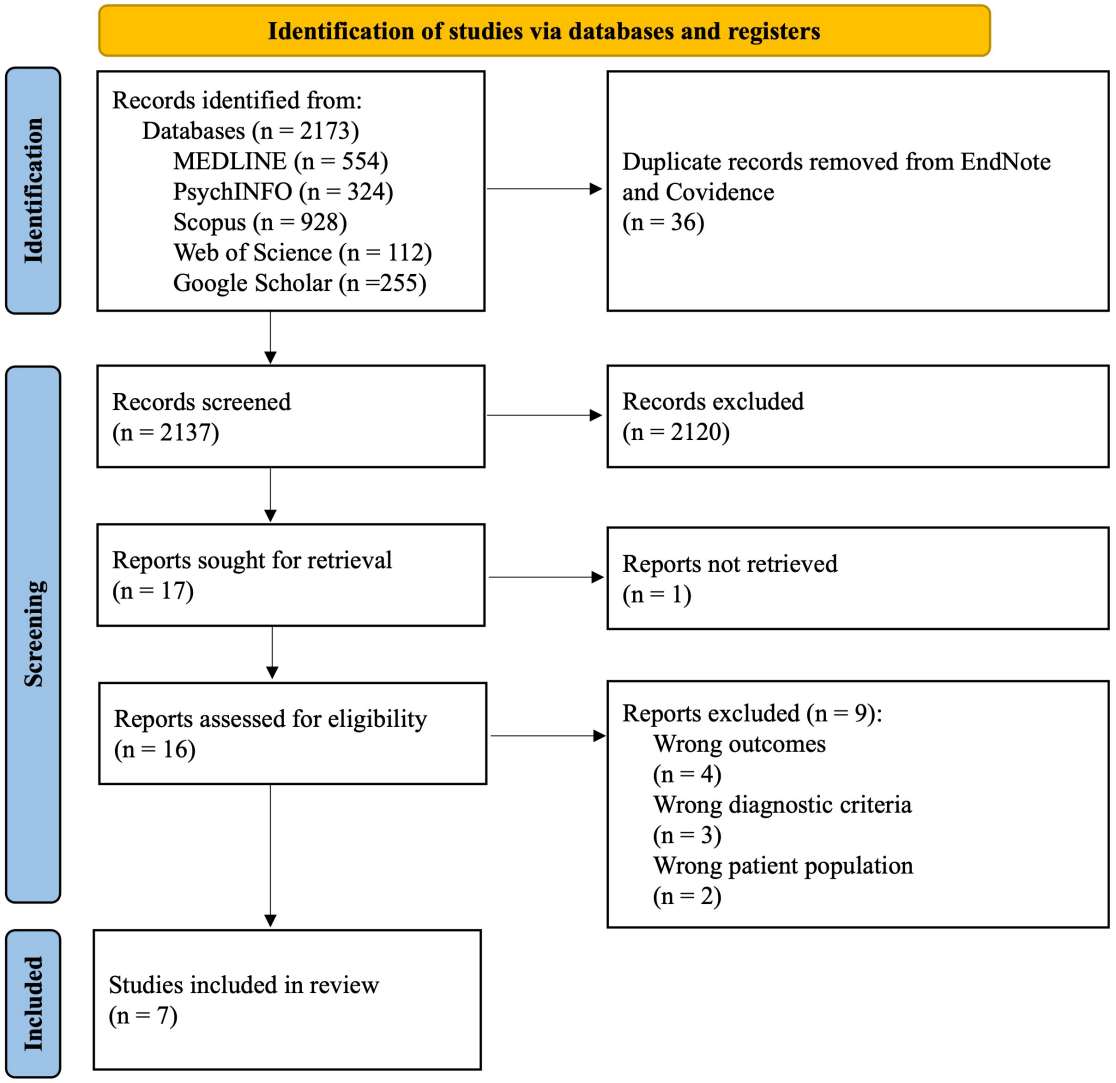
PRISMA flow diagram for the selection of review articles.

A total of 2,173 potential records were identified, of which 36 were duplicate articles that were subsequently removed (see Supplementary Table 1 for details regarding the original and updated searches). Titles and abstracts of 2,137 articles were then screened. For 27 studies (1.3% of reviewed sample), disagreements regarding eligibility were resolved between screening raters. Of the potentially eligible articles, 17 articles met inclusion criteria; however, one study was unable to be accessed/retrieved and was therefore not included in the full-text review. Sixteen studies therefore underwent full-text review. Disagreements for study inclusion were resolved between study raters for three studies (18.8% of reviewed sample). Seven studies were selected to be included in the final review (30–32, 35–38), including five cohort studies and two case–control studies (no clinical trials were identified). A detailed flowchart of the study selection process (PRISMA) for the final search can be found in Figure 1.

### 3.2. Cohort studies

#### 3.2.1. Characteristics of included studies

Five cohort studies met inclusion criteria (30, 32, 35–37). All studies had a retrospective study design, with two studies (30, 35) additionally having a matched-cohort design. Exposure and outcomes were operationalized using ICD codes (versions: ICD-7, ICD-8, ICD-9, ICD-9-clinical modification, and ICD-10), obtained through electronic health records in all studies. Exposure (i.e., ADHD status) was most often defined using the ICD-9 code 314 “Hyperkinetic syndrome of childhood” or 314.01 “Attention deficit disorder with hyperactivity” and the ICD-10 code F90 “Attention-deficit hyperactivity disorders.” Two studies used patient/population register data from Sweden (36, 37), two studies used healthcare data from the United States (32, 35), and one study used health insurance data from Taiwan (30). For more detailed information about the study including aims, population selected, and inclusion/exclusion criteria, please see Supplementary Table 1.

Participants across the studies ranged from younger adults (30’s) to older adults (60’s). Only one study (35) reported education years for the exposed and non-exposed groups (not shown in Table 1); no study reported race or ethnicity of participants. Detailed characteristics for each study, stratified by outcomes (i.e., neurodegenerative disease types), are described in Table 1. The following subtypes of outcomes were reported in the studies: all-cause dementia (30, 36, 37), AD (30, 32), PD (35, 36) and LBD (32), VaD (30), and MCI (37). Across studies, the prevalence for each type of dementia across all participants in each study was somewhat varied: all-cause dementia, <0.001 to 4.37%, AD 0.78%, PD 0.08 to 0.26%, VaD 0.44%, MCI 0.65%. Prevalence of ADHD in adults was relatively low across studies, ranging generally from 0.19 to 1.29%, but as high as 16.7% in one study (35). One study (32) did not report numbers of participants included in the analyses (and we were unable to reach the authors), so prevalence for both exposure and outcome measures could not be calculated.

**Table 1:** Summary of findings from observational cohort studies for dementia risk in both exposed (ADHD) and unexposed (control) groups, stratified by dementia type.

Study	Total, *N*	Age*	Sex, female (%)	Outcomes, *N*	Ratio (crude)	Ratio (adjusted for sex/birth year)	Ratio (adjusted for other covariates)	Other adjustment	Follow-up years	Exposure Definition	Outcome definition
All-cause dementia											
Dobrosavljevic et al. (37)	3,588,910	63 (56–70)^a^	49.30% ^a^	55,194	–	HR 2.93 [2.15–4.00]	HR 0.98 [0.72–1.34]	Sex, birth year, educational attainment, metabolic disorders, sleep disorders, head injuries, psychiatric disorders, and other developmental disorders	13	ICD-9 code 314, ICD-10 code F90	ICD-8 codes 290, 293.0, 293.1; ICD-9 codes 290A/B/X, 290E, 290 W, 294B, 331A, 331B/C/X; ICD-10 codes F00, F01, F02, F02.1, F02.2, F02.3, F02.4, F02.8, F03, F05.1, G30, G31.1, G31.8
Exposed	6,753	55 (52–60) ^a^	45.80% ^a^	100							
Unexposed	3,582,157	63 (56–70)	49.30%	55,094							
DuReitz et al. (36)	4,789,799	47 (18-81)^b^	49%	20,729	–	OR 2.44 [1.86–3.19]			–	ICD-9 code 314, ICD-10 code F90 OR ATC N06BA01, N06BA02, N06BA09, 06BA12	ICD-8 codes 29,000, 29,010, 29,011, 29,019, 29,300, 2,931; ICD-9 codes 290A, 290B, 290E, 290 W, 290X, 294B, 331A, 331B, 331C, 331X; ICD-10 codes G30, G31.1, G31.8A, F00-F03, F05.1
Exposed	61,960	–	–	73							
Unexposed	4,727,839	–	–	20,656							
Tzeng et al. (30)	2,700	28.2 (12.4)	27.56%	118	HR 3.418 [2.289–5.106]		HR 4.008 [2.526–6.361]	Age group, sex, comorbidities, geographical area of residence, urbanization level of residence, and monthly income	10	ICD-9-CM code 314	ICD-9-CM codes 290.0, 290.10, 290.11, 290.12, 290.13, 290.20, 290.21, 290.3, 290.41, 290.42, 290.43, 290.8, 290.9, 331.0
Exposed	675	–	27.56%	37							
Unexposed	2,025	–	27.56%	81							
Alzheimer’s disease/dementia											
Fluegge and Fluegge (32)				162	–		IRR 0.99 [0.91–1.06]	Other mental health hospitalizations, diabetes, and obesity	10	ICD-9 code 314.01	ICD-9 code 331.0
Exposed	–	–	–	–							
Unexposed	–	–	–	–							
Tzeng et al. (30)	2,700	28.2 (12.4)	27.56%	21	–		HR 0.524 [0.061–4.526]	Age group, sex, comorbidities, geographical area of residence, urbanization level of residence, and monthly income	10	ICD-9-CM code 314	ICD-9-CM codes 290.0, 290.10, 290.11, 290.12, 290.13, 290.20, 290.21, 290.3, 331.0
Exposed	675	–	27.56%	2							
Unexposed	2,025	–	27.56%	19							
Lewy body disease											
Curtin et al. (35)	190,559	28.2 (12.4)	42.7%	152 PD	HR 2.9 [2.2–3.9]		HR 2.6 [1.8–3.7]	Sex, age, race/ethnicity, psychotic conditions, tobacco use, and an interaction of psychotic conditions and ADHD	21	ICD-9 codes 314.00, 314.01, 314.1, 314.2, 314.8, 314.9	ICD-9 codes 332.0, 332.1, 333.0, 333.1
Exposed	31,769	–	42.7%	56 PD							
Unexposed	158,790	–	42.7%	96 PD							
Du Reitz et al. (36)	4,789,799	47 (18-81)^b^	49%	12,569 PD	–	OR 1.50 [1.08–2.09]			–	ICD-9 code 314, ICD-10 code F90 OR ATC N06BA01, N06BA02, N06BA09, 06BA12	ICD-8 codes 34,200, 34,208, 34,209; ICD-9 codes 332.0, 332.1, 333.0; ICD-10 codes G20, G21·2, G21·3, G21·8, G21·9, G23·1, G23·2, G23·8, G23·9, G25·9
Exposed	61,960	–	–	47 PD							
Unexposed	4,727,839	–	–	12,522 PD							
Fluegge and Fluegge (32)				162 LBD	–		IRR 1.06 [0.95–1.18]	Other mental health hospitalizations, diabetes, and obesity	10	ICD-9 code 314.01	ICD-9 code 331.82
Exposed	–	–	–	–							
Unexposed	–	–	–	–							
Vascular dementia											
Tzeng et al. (30)	2,700	28.2 (12.4)	27.56%	12	–		HR 6.284 [2.710–25.853]	Age group, sex, comorbidities, geographical area of residence, urbanization level of residence, and monthly income	10	ICD-9-CM code 314	ICD-9-CM code 290.4
Exposed	675	–	27.56%	5							
Unexposed	2,025	–	27.56%	7							
Mild cognitive impairment											
Dobrosavljevic et al. (37)	3,588,910	63 (56–70) ^a^	49.30% ^a^	23,507	–	HR 6.39 [5.11–8.00]	HR 1.71 [1.36–2.15]	Educational attainment, metabolic disorders, sleep disorders, head injuries, psychiatric disorders, and other developmental disorders	13	ICD-9 code 314, ICD-10 code F90	ICD-10 code F06.7
Exposed	6,753	55 (52–60) ^a^	45.80% ^a^	142							
Unexposed	3,582,157	63 (56–70)	49.30%	23,365							

– unknown/not reported, *Age is given as Mean (Standard Deviation) or Median (Interquartile Range). ^a^Characteristics reported reflect the inclusion of ADHD participants in the total sample who were diagnosed solely based on intake of ADHD medication, while the ratios reported reflect only the inclusion of ADHD participants who were diagnosed based on ICD codes. ^b^Age is given as Mean (Range).

ADHD, Attention-Deficit Hyperactivity Disorder; ATC, Anatomical Therapeutic Chemical Classification; CM, Clinical Modification; HR, Hazard Ratio; ICD, International Classification of Diseases; IRR, Incidence Rate Ratio; LBD, Lewy Body Diseases; OR, Odds Ratio; PD, Parkinson’s Disease.

#### 3.2.2. Risk of bias assessment

The potential risk of bias, quantified using the NOS for cohort studies, is shown in Table 2. Three studies were rated as being at overall low risk of bias (30, 35, 37), and two studies were rated as having a medium risk of bias (32, 36). Although all studies were not biased in their ascertainment of exposure (record linkage using ICD codes is considered sufficient), all were biased in their representativeness of the exposed cohort. Furthermore, despite using epidemiological data, only two studies made statements regarding follow-up of cohorts (35, 37).

**Table 2:** Risk of bias assessment using the Newcastle Ottawa Scale for cohort studies, adapted with permission from Becker et al. (16).

	Representativeness of the exposed cohort	Selection of the non-exposed cohort	Ascertainment of exposure	Demonstration that outcome of interest was not present at start of study	Comparability of cohorts on the basis of the design or analysis	Assessment of outcome	Was follow-up long enough for outcomes to occur	Adequacy of follow up of cohorts	Overall quality
Curtin, et al. (35)	Included several non-ADHD hyperkinetic syndromes	Drawn from the same community as the exposed cohort 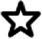	ICD 9-CM codes linked to Utah Population Database 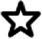	Patients were excluded if BG&C disorders were present prior to an index ADHD diagnosis or before age 21 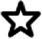	Matched on sex and birth year; analyses controlled for race, ethnicity, psychotic conditions and tobacco use 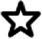 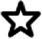	ICD 9-CM codes linked to Utah Population Database 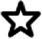	1996 to 2016 (median follow-up was 21 years) 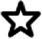	2.5% cases lost to follow-up vs. <1% controls; statistical models included a competing risk of death 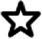	8/9
Dobrosavljevic et al. (37)	Included several non-ADHD hyperkinetic syndromes	Drawn from the same community as the exposed cohort 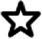	ICD-9 and ICD-10 codes and medication prescriptions from multiple Swedish population-based registers 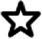	Diagnosis of MCI or dementia must have been after age 50, but unclear whether ADHD diagnosis may have come before or after dementia diagnosis	Adjusted for sex and birth year, covariates: educational attainment, metabolic disorders, sleep disorders, head injuries, psychiatric disorders, and other developmental disorders 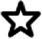 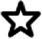	ICD-7, ICD-8, ICD-9, and ICD-10 codes from multiple Swedish population-based registers 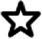	Follow-up period was at least 13 years after age 50 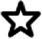	6% of ADHD participants lost vs. 9% of controls lost 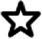	7/9
Du Rietz et al. (36)	Included several non-ADHD hyperkinetic syndromes or individuals prescribed ADHD medication	Siblings, half-siblings. And family members; Drawn from the same community as the exposed cohort 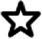	ICD-9 and ICD-10 codes linked to the National Patient Register 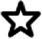	Not stated	Stratified by sex, and birth year of relatives to adjust for follow-up lengths 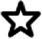 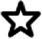	ICD-9 and ICD-10 codes linked to the National Patient Register 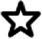	Not stated	No information provided	5/9
Fluegge and Fluegge (32)	Only considered ADD with hyperactivity (not inattentive presentation); only considered *hospitalization* for ADHD	Drawn from the same community as the exposed cohort 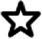	ICD 9-CM codes linked to the Healthcare Cost and Utilization Project 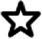	Not stated	Not stated whether cohorts were comparable; analyses adjusted for age, diabetes and obesity 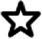 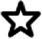	ICD 9-CM codes linked to the Healthcare Cost and Utilization Project 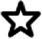	Ten-year lagged measure 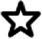	No information provided	6/9
Tzeng et al. (30)	Included several non-ADHD hyperkinetic syndromes; exposed cohort restricted to inpatients, or those with ≥3 outpatient visits within 1 year	Drawn from the same community as the exposed cohort 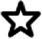	ICD 9-CM codes linked to the National Health Insurance Program 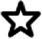	Participants excluded if dementia was present before tracking began or before an ADHD diagnosis 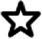	Matched on sex, age, geographic area and urbanization of residence, comorbidities, and income 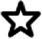 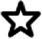	ICD 9-CM codes linked to the National Health Insurance Program 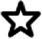	2000–2010 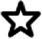	No information provided	7/9

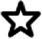
 Indicates a high-quality choice, with a maximum of one star in all categories except “Comparability” which has a two-star maximum.

AD, Alzheimer’s Disease/Dementia; ADHD, Attention-Deficit Hyperactivity Disorder; BG&C, Basal Ganglia and Cerebellar Disorders; CM, Clinical Modification; HCUP, Healthcare Cost and Utilization Project; ICD, International Classification of Diseases.

### 3.3. Case–control studies

#### 3.3.1. Characteristics of included studies

Two case–control studies met inclusion criteria (31, 38); one study used health insurance data from Taiwan (38), and the other hospital records from Argentina (31). Both studies matched cases and controls: 1:1 by sex, age, and index date (38), and 1:2 by sex, age, geographic area, and education (31). For detailed information about the study including aims, population selected, and inclusion/exclusion criteria, please see Supplementary Table 1.

The two case–control studies reported on AD (31), PD (38), and DLB (31) as outcomes. Exposure (i.e., ADHD status) was defined using the DSM-IV criteria or the ICD-9 code 314 “Hyperkinetic syndrome of childhood.” Detailed characteristics for each study, stratified by outcomes, are described in Table 3. Ages of participants were similar in both studies (means between 70 and 75 years), while the percentage of females differed (67% versus 49%). Only one study (31) reported education years for the cases and controls (not reported in Table 3); neither study reported race or ethnicity of participants.

**Table 3:** Summary of findings from case–control studies, grouped by dementia type.

Study	Total, *N*	Age, mean (standard deviation)	Sex, female (%)	ADHD, *N*	Matching variables	Ratio (crude)	Ratio (adjusted for other)	Other adjustment	Follow-up years	Exposure definition	Outcome definition
Case–control* studies of Alzheimer’s disease risk											
Golimstok et al. (31)	400				1:2, age, sex, geographic area of residence, years of education	OR 1.1 [0.7–1.5]	–	–	5	DSM-IV criteria; Wender Utah Rating Scale	NINCDS-ADRDA criteria (39)
Case	251	74.2 (7.1)	68.1%	38							
Control	149	74.1 (8)	66.7%	22							
Case–control* studies of Lewy body disease risk											
Fan et al (38)	21,452			19	1:1 by age, sex, and index date	OR 2.80 [1.01–7.78]	OR 3.65 [2.26–10.50]	Sex, age, and Charlson Comorbidity Index	14	ICD-9 code 314	ICD-9 code 332
Case	10,726 PD	70.1 (14.0)	49.1%	14							
Control	10.726	70.0 (14.1)	49.1%	5							
Golimstok et al. (31)	258				1:2, age, sex, geographic area of residence, years of education	OR 5.1 [2.7–9.6]	–	–	5	DSM-IV criteria; Wender Utah Rating Scale	Consensus criteria (40)
Case	109 DLB	75.1 (7.4)	67.4%	52							
Control	149	74.1 (8)	66.7%	22							

– unknown/not reported. *In case control studies individuals are sampled at the start of the study based on whether they have the outcome of interest (i.e., neurodegeneration/dementia) or not.

ADHD, Attention-Deficit Hyperactivity Disorder; CDR, Clinical Dementia Rating Scale; DLB, Dementia with Lewy Bodies; DSM, Diagnostic and Statistical Manual of Mental Disorders; ICD, International Classification of Diseases; MMSE, Mini Mental State Examination; NINCDS-ADRDA, National Institute of Neurological and Communicative Diseases and Stroke/Alzheimer’s Disease and Related Disorders Association; OR, Odds Ratio.

#### 3.3.2. Risk of bias assessment

The potential risk of bias, quantified using the NOS for case–control studies, is shown in Table 4. One study was rated as being at overall low risk of bias (31), and the other as having a medium risk of bias (38). For both, it was unclear whether the control cases constituted a hospitalized sample, so the selection of controls was rated as inadequate. Only Golimstok and colleagues (31) used adequate definitions for both outcomes and exposures.

**Table 4:** Risk of bias assessment using the Newcastle Ottawa Scale for case–control studies, adapted with permission from Becker et al. (16).

	Is the case definition (ND outcome) adequate?	Representativeness of the cases	Selection of controls	Definition of controls	Comparability of groups	Ascertainment of exposure	Same method of ascertainment for cases and controls?	Non-response rate	Overall quality
Fan et al. (38)	ICD 9-CM code of PD with ≥3 outpatient visits or hospital admissions and receiving PD medication	Yes 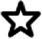	Same sample as cases, but unclear if controls constitute a hospitalized sample	Controls were ‘subjects without PD’ 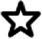	Groups matched on sex, age, and index date; analyses used Charlson Comorbidity Index 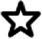 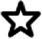	ICD-9-CM code of 314.0 (ADD with and without hyperactivity)	Yes 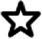	Same in both groups (0%) 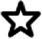	6/9
Golimstok et al. (31)	Probable AD based on NINCDS/ADRDA criteria. DLB based on consensus criteria; diagnoses based on several sources 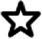	Yes 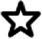	Same sample as cases, but unclear if controls constitute a hospitalized sample	No history of dementia or neurological disease 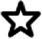	Groups matched on sex, age, geographic area of residence and education 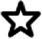 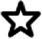	DSM-IV criteria ascertained by clinician blind to case/control status 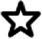 + Retrospective self-report	Yes 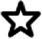	Same in both groups (0%) 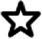	8/9

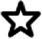
 Indicates a high-quality choice, with a maximum of one star in all categories except “Comparability” which has a two-star maximum. AD, Alzheimer’s Disease; ADD, Attention Deficit Disorder; ADHD, Attention-Deficit Hyperactivity Disorder; CM, Clinical Modification; DSM-IV, Diagnostic and Statistical Manual of Mental Disorders Fourth Edition; HCUP, Healthcare Cost and Utilization Project; ICD, International Classification of Diseases; LBD, Lewy Body Diseases; PD, Parkinson’s Disease.

### 3.4. Meta-analysis and subgroup analyses

The planned meta-analysis was not feasible as there was an insufficient number of homogeneous studies to calculate a pooled risk ratio. Of all seven studies, only four studies reported crude ratios (of which two were case–control and two were cohort studies) and all calculated different ratios for risk (three studies calculated hazard ratios, three studies calculated odds ratios, and one study calculated an incidence rate ratio). Furthermore, each study controlled or covaried for different factors: two adjusted for demographics (sex and birth year) (36, 37), while all others covaried for sex and birth year/age along with additional covariates (ranging from three to seven additional covariates) (30–32, 35, 38). In theory, a meta-analysis can be performed on two or more studies (24, 41). However, with only a few studies with very different characteristics, any kind of synthesis is untenable and does not yield a meaningful summary estimate of the effect in most cases (41, 42). More importantly, parameter estimation is likely to be poor with so few studies, leading to highly questionable findings (41). Lastly, the planned subgroup analyses were also not feasible as there were either not enough studies per dementia subtype, or studies were too heterogeneous in the ratios used and covariates included to be able to synthesize the data.

## 4. Discussion

This is the first systematic review examining the risk of incident neurodegenerative disease or dementia associated with ADHD. From the literature search, only a few cohort and case–control studies were identified that examined this association, all with different populations and methodologies. Although the study differences prevented data synthesis, the individual results tentatively suggest a link between ADHD in adults and development of neurodegeneration or dementia.

Results show that ADHD is differentially associated with all-cause and subtypes of dementia. Interestingly, the highest ratio was found for VaD, where people with ADHD had a 6-fold higher risk than controls after adjustment for covariates. VaD is defined as severe cognitive impairment compromising daily functioning and evidence of cerebrovascular disease on imaging (43). Adults with ADHD have notably worse cardio- and cerebrovascular health than adults without ADHD (44–46), which may lead to impaired brain health and subsequently higher risk of developing vascular dementia. Incidentally, the study examining risk of VaD also controlled for vascular risk factors (among others) including diabetes, hypertension, coronary artery disease, and stroke (30). This suggests that this risk remains despite the presence of these risk factors.

People with ADHD also have a high risk of developing LBD, as shown by the cohort studies revealing an incidence rate ratio of 1.06 for dementia with Lewy bodies, and a 1.5–2.6 times higher risk of developing PD compared to controls. The case–control studies showed people with dementia with Lewy bodies were 5.1 times more likely to have been diagnosed with ADHD than controls, and people with PD were 3.7 times more likely. This effect was independent of age and sex. ADHD and LBD, specifically PD, have been previously hypothesized to be linked pathophysiologically through the dopaminergic system (17). While ADHD involves a dysregulation of the dopaminergic system (47), the symptoms of PD are caused by degeneration of the dopaminergic system (48). One study suggested that low dopamine levels and abnormal maturation of the dopaminergic system in people with ADHD could constitute a risk for developing PD in later life (49), while another postulated ADHD and PD may be related as two points on a continuum (31). Studies examining genetics have, however, been unable to find links or causality between PD and ADHD (50, 51), suggesting there may be other causes for the association. It has been suggested that stimulants, the most common medications for ADHD, may explain the association between ADHD and PD. Research in animal and human studies suggest that stimulants may have toxic effects on dopaminergic neurons and result in dysfunctional dopamine regulation and transport (17). On the contrary, a previous review found that stimulant use does not increase risk for PD (52), and a recent study with older adults with ADHD demonstrated that participants who had taken prescription stimulants had a *reduced* risk for developing PD relative to participants who had not been prescribed stimulants (53). In this current review, only one study accounted for medication (35), finding that the adjusted hazard ratios for developing PD in people with ADHD with and without stimulants were 3.9 and 2.3, respectively. However, they selected participants based on known use of stimulants, so effects of dosage or length of stimulant use was not factored in. More studies are needed to adequately determine the link between ADHD and risk of PD or DLB, as well as understanding the role of dopamine in this association. Future studies should also include medication use and examine how dosage and length of use can affect future risk of dementia or neurodegenerative diseases.

Relative to other types of neurodegenerative disease, there was a somewhat lower risk for development of MCI in people with ADHD, and associations between ADHD and AD were not significant in any cohort or case–control studies. It is possible that MCI risk associated with ADHD may be explained by the similarities of the two disorders. Research shows that symptoms of ADHD (e.g., difficulty paying attention, difficulty inhibiting impulsivity, forgetfulness, absent-mindedness) may be misinterpreted as signs of MCI or early AD (54). Although most studies clarified that ADHD must have been diagnosed before presence of neurocognitive disorders or dementia, this warrants further investigation.

All-cause dementia was the most variable in terms of risk, where adjusted hazard ratios ranged from 0.98–4.01. It has been suggested people with ADHD have an increased risk of overall health problems, and that ADHD itself leads to the development of factors or disorders that can compromise health and which are, in and of themselves, risk factors for dementia (54, 55). Almost 80% of adults with ADHD have a comorbid psychiatric disorder, including anxiety (47%) or mood disorders such as depression (38%) and bipolar disorder (15%) (56). As previously stated, these factors are themselves associated with a higher risk of developing dementia and may therefore also lead to increased cognitive dysfunction or dementia in adults with ADHD (57). In addition, adults with ADHD have higher rates of smoking (58, 59) and vascular factors such as obesity, hypercholesteremia, and hypertension (44, 60). Presence of these factors in mid-life has also been shown to increase risk of dementia (9). It is possible that the direct effect of ADHD on dementia risk is being masked in part by these comorbid psychiatric disorders or vascular factors. Three studies controlled for comorbid mental/psychiatric disorders (among other covariates) (30, 35, 37), and one additional study controlled for mental health hospitalizations which were not further defined in the study (however it was unclear whether this was a part of patient selection) (32). Three studies controlled for comorbid cerebrovascular diseases and vascular factors, among others (30, 32, 37, 38). Controlling for these factors often significantly attenuated or eliminated the effect of ADHD on later-life dementia: for example, Dobrosavljevic and colleagues (37) found that after controlling for educational attainment, metabolic disorders, sleep disorders, head injuries, psychiatric disorders, and other developmental disorders, the hazard ratio for developing all-cause dementia dropped from 2.93 to 0.98 and for MCI dropped from 6.39 to 1.71 in people with ADHD. The authors additionally noted that the relationship between ADHD and both dementia and MCI was substantially attenuated after controlling for only psychiatric disorders, but less impacted when controlling only for metabolic disorders (37). Interestingly, Tzeng and colleagues (30) found a stronger risk of all-cause dementia in people with ADHD when controlling for age group, sex, comorbidities (including a number of psychiatric disorders and vascular factors), geographical area of residence, urbanization level of residence, and monthly income (crude hazard ratio 3.15, adjusted hazard ratio 4.01). It seems therefore unclear whether controlling for vascular and comorbid psychiatric disorders attenuates or increases the risk of later dementia. It is important for future studies to take this into consideration and control for the possibility of mediating or moderating relationships of comorbid disorders on the relationship between ADHD and dementia.

It should be noted that the prevalence of ADHD in all studies (from 0.19–16.7%) is markedly different than the global prevalence which is approximately 3% in adults (61, 62). This is a surprising finding as almost all studies used electronic health data from nationwide population or health registers, with one study even including medication prescriptions for ADHD in their diagnostic criteria (36). Arguably, use of healthcare and register data enables researchers to examine population-level data, usually collected over long periods of time and linked through multiple registers or databases, leading to less chance of bias (63, 64). However, ADHD in adults is often underdiagnosed (65, 66) or misdiagnosed as cognitive decline (54), which may suggest that some people with ADHD may have been mislabeled as controls in the selected studies. Additionally, we did not limit whether adults with ADHD had to have received the diagnosis as a child. In the studies, inclusion criteria regarding diagnosis timing were varied: participants either must have had a diagnosis as an adult (30, 31, 36), had a diagnosis of ADHD at any point in their life (35, 37, 38), or it was unclear when participants received their diagnosis (32). Including only participants who had a verified diagnosis as an adult may have biased the sample toward more severe cases of ADHD, as persons with remitted ADHD – which account for 50% of cases (21) – are unaccounted for in the included studies. Furthermore, each study used widely varying inclusion criteria: two studies required participants to have had a certain number of in- or outpatient visits for the diagnosis to be considered valid (30, 38), and one study required all participants to have a sibling (either full or maternal half-sibling) (36). This may have also biased the samples of ADHD participants to more severe cases, inadequately representing the population at risk and contributing to the very low prevalence of adult ADHD in these samples.

### 4.1. Meta-analysis limitations and recommendations

While the individual results suggest there is a link between ADHD and development of a subsequent neurodegenerative disease/dementia, we were unable to determine the pooled risk of developing a neurodegenerative outcome due to the marked heterogeneity of the studies. The main challenge to the synthesis of data was the use of different ratios to assess risk – three studies calculated hazard ratios, three studies calculated odds ratios, and one study calculated an incidence rate ratio – and not all studies reported crude or unadjusted ratios. It will be essential for future studies to report crude ratios along with any adjusted analyses to facilitate data synthesis for future meta-analyses.

Another challenge to data synthesis was the definition of exposures and outcomes used in each study. The definitions of both ADHD and neurodegeneration, despite being mostly classified by ICD codes, were different across studies. This has also been extensively discussed in our previous critical review of these studies (16). For example, only one case–control study had an adequate definition of exposures (and was subsequently the only study with a low risk of bias for representativeness of the exposed cohort). We have also previously commented on the use of the ICD-9 and ICD-10 codes for diagnosing ADHD in these studies (16). Most notably, differing inclusion of hyperactivity or hyperkinetic disorders in the diagnosis of ADHD may account for differences in prevalence and dementia risk.

Lastly, as discussed earlier, it is important to control for known risk factors for dementia and those that may be mediating or confounding the relationship between ADHD and dementia risk, such as comorbid psychiatric disorders or vascular factors. It is also important to include demographic factors including age, sex, race/ethnicity, as well as socioeconomic factors such as education, income level, and relationship/social support. While age is the strongest risk factor for dementia (6, 8), sex is also an important factor and risk depends on the subtype of dementia studied (9). Females have a higher risk of developing AD and a slightly higher risk for DLB (67, 68), while males have a higher risk of developing VaD and PD (69, 70). Furthermore, higher educational attainment reduces the risk of dementia in later life (9), and race/ethnicity may affect dementia risk: in the United States, Black and Hispanic people had higher risk of dementia when compared to White people (71). These factors are important to consider when researching people with ADHD. Males are two- to ten-times more commonly diagnosed with ADHD and have a higher risk of developing a neurodevelopmental disorder than females (72, 73), and ADHD remains underdiagnosed in females (55). Furthermore, adults with ADHD tend have lower educational attainment (74) and income levels (75), as well as poor social adjustment including higher rates of divorce or relationship dissatisfaction (76–78). Both social isolation and worse educational attainment have been shown to increase subsequent risk of dementia (9). Less is known about how race and ethnicity affect the diagnosis of ADHD, but minority groups, when compared with White persons, are less likely to receive or get assessment for an ADHD diagnosis (79, 80). While age and sex are readily available using electronic health records, records often do not adequately capture race, ethnicity, and the socioeconomic factors (81, 82). Indeed, while all but one study adjusted analyses for, or matched participants on, sex and age or birth year, no study used race or ethnicity in their analyses. Tzeng and colleagues (30) controlled for income levels and urbanization level of residence, and only Dobrosavljevic and colleagues (37) included educational attainment in their adjusted analyses. Additionally, Golimstok and colleagues (31) matched cases and controls on geographic area of residence and education. As these studies included these factors along with other covariates in their analyses, it is not possible to determine the individual effect of each covariate on dementia risk.

Overall, the challenges to performing a meta-analysis highlight the need for improved methodology in assessing the link between ADHD and neurodegeneration, as well as improved study reporting.

### 4.2. Limitations

Our strict inclusion/exclusion criteria led to the exclusion of half the studies that were identified through abstract screening. ADHD must have been diagnosed using standardized (i.e., using DSM or ICD) criteria, which led to exclusion of three studies: two used genetic scores (polygenic risk scores) to determine ADHD status and one study used a retrospective questionnaire for ADHD symptoms. The outcomes must have been defined by standardized or consensus criteria, which, for example, led to exclusion of one study examining MCI defined using only a screening battery. We justified this *a priori* to ensure that diagnoses of both exposure and outcomes were valid and reliable.

## 5. Conclusion

This is the first systematic review to examine the relationship between adult ADHD and future development of a neurodegenerative disease or dementia. Our review provides preliminary results that a diagnosis of ADHD may be a risk factor for the later development of a neurodegenerative disease or dementia. The mechanism of how or why ADHD is associated with an increased risk of developing a neurocognitive disorder is still unclear and should be explored in future studies. Due to the heterogeneity of studies included, no meta-analysis of data was possible, and we were unable to determine the pooled risk for developing a neurodegenerative disease or dementia in people with ADHD. This highlights the need for more stringent and well-defined studies, and we advocate for improvements in study methodology and statistical analyses to further advance this research.

## Data availability statement

The original contributions presented in the study are included in the article/Supplementary material, further inquiries can be directed to the corresponding author.

## Author contributions

SB and BC conceived the research question and designed the study. PT conducted the literature search. SB, MC, PT, DS, and BC screened titles and abstracts for inclusion in the study. SB and BC screened full texts for final inclusion into the study, extracted data, and rated each manuscript for quality. SB wrote the first draft of the manuscript. MC, PT, DS, and BC contributed substantially to drafting the article and revising it critically for intellectual content. All authors have read and agreed to the published version of the manuscript.

## Funding

This review received no specific grant from any funding agency. SB receives salary support from a Canadian Institutes of Health Research Fellowship. MC receives salary support from a Mathison Centre Postdoctoral Fellowship and an O’Brien Institute for Public Health Postdoctoral Scholarship. PT receives salary support from the Alzheimer’s Association. DS receives grant funding from the Canadian Institutes of Health Research – Canadian Consortium on Neurodegeneration in Aging, the Alzheimer’s Association, and the University Health Foundation – Alberta Roche Collaboration in Health. BC’s research program is supported by the Canada Research Chairs Program.

## Conflict of interest

The authors declare that the research was conducted in the absence of any commercial or financial relationships that could be construed as a potential conflict of interest.

## Publisher’s note

All claims expressed in this article are solely those of the authors and do not necessarily represent those of their affiliated organizations, or those of the publisher, the editors and the reviewers. Any product that may be evaluated in this article, or claim that may be made by its manufacturer, is not guaranteed or endorsed by the publisher.

## Footnotes

^1^https://www.crd.york.ac.uk/prospero
