# Supplementary material for: Risk of neurodegenerative disease or dementia in adults with attention-deficit/hyperactivity disorder: a systematic review
Source: Front Psychiatry. 2023 Aug 17;14:1158546. doi: 10.3389/fpsyt.2023.1158546 (PMC10469775; doi:10.3389/fpsyt.2023.1158546)
Supplement: Supplementary file 1 [file Data_Sheet_1.docx]

Supplementary Material

Risk of neurodegenerative disease or dementia in adults with attention-deficit/hyperactivity disorder: a systematic review

Sara Becker, Mohammad Chowdhury, Pattara Tavilsup, Dallas Seitz, Brandy L. Callahan*

*** Correspondence:** Brandy Callahan: brandy.callahan@ucalgary.ca

# Supplementary Data: Search Strategy

Search Terms

| **Databases** | **Results (September 2022)** | **Updated Results (May 2023)** |
| --- | --- | --- |
| Ovid MEDLINE | 491 | 554 |
| Ovid PsycINFO | 297 | 324 |
| Scopus | 816 | 928 |
| Web of Science | 103 | 112 |
| Google Scholar | 113 | 255 |
| Search Total | 1820 | 2173 |
| Duplicates removed in EndNote and Covidence | 238 | 1582 removed because they were screened previously; 36 additional duplicates removed |
| Total references in Covidence | 1582 | 555 |
| Total references screened | 2137 | |

**Phase 1 – Sources to search:**

Databases and resources to be searched:

- Electronic databases: Ovid MEDLINE; Ovid PsycINFO; Scopus; Web of Science; Google Scholar
- Reference lists of eligible articles will be hand searched for further relevant studies
- The search was conducted between August 23 and September 27, 2022 and updated May 17, 2023, with the guidance of Caitlin McClurg, an associate librarian, University of Calgary

**Phase 2 -- Refine the search:**

Keywords / Index terms (MeSH):

- Attention Deficit Disorders with Hyperactivity
- ADHD
- Attention Deficit Hyperactivity Disorder
- Hyperkinetic Syndrome
- Syndromes, Hyperkinetic
- Attention Deficit-Hyperactivity Disorder
- Deficit-Hyperactivity Disorder, Attention
- Disorder, Attention Deficit-Hyperactivity
- ADDH
- Attention Deficit Disorder
- Deficit Disorder, Attention
- Disorder, Attention Deficit
- Neurodegenerative Disease
- Neurologic Degenerative Disease
- Nervous System Degenerative Disease
- Neurodegenerative Disorder
- Neurologic Degenerative Conditions
- Degenerative Neurologic Disorder
- Neurologic Disorder, Degenerative
- Degenerative Diseases, Spinal Cord
- Degenerative Diseases, Central Nervous System
- Dementia
- Dementia, Vascular
- Dementia, Multi-Infarct
- [Synucleinopathies](https://www.ncbi.nlm.nih.gov/mesh/2031016)
- Lewy Body Disease
- Multiple System Atrophy
- Parkinson Disease
- Parkinsonian Disorders
- Basal Ganglia Diseases
- Tauopathies
- Alzheimer Disease
- [Corticobasal Degeneration](https://www.ncbi.nlm.nih.gov/mesh/2101076)
- Diffuse Neurofibrillary Tangles with Calcification
- Supranuclear Palsy, Progressive
- TDP-43 Proteinopathies
- Amyotrophic Lateral Sclerosis
- Frontotemporal Lobar Degeneration
- Frontotemporal Dementia
- Mild Cognitive Impairment
- Cognitive Impairment, Mild
- Impairment, Mild Cognitive
- Mild Cognitive Impairments
- Mild Neurocognitive Disorder
- Disorder, Mild Neurocognitive
- Mild Neurocognitive Disorders
- Neurocognitive Disorder, Mild
- Cognitive Decline
- Decline, Cognitive
- Prevalence
- Prevalence case control study
- Incidence
- Risk
- Case-Control Studies
- Cohort studies

**Phase 3 -- Comprehensive search strategy**

**Note:** The search results presented below reflect the updated search conducted on May 17, 2023.

1. *Medline (Ovid) (May 16th, 2022)*

Ovid MEDLINE(R) and Epub Ahead of Print, In-Process, In-Data-Review & Other Non-Indexed Citations and Daily <1946 to May 16, 2023>

| **Concept** | **Number** | **Keywords and MeSH** | **Results** |
| --- | --- | --- | --- |
| Attention-deficit/hyperactivity disorder | 1 | exp *Attention Deficit Disorder with Hyperactivity/ or Attention Deficit Disorder with Hyperactivity.tw,kf. or (addh or adhd or attention deficit disorder* or attention deficit disorder* with hyperactivity or attention deficit hyperactivity disorder* or attention deficit-hyperactivity disorder* or brain dysfunction, minimal or deficit disorder*, attention or deficit-hyperactivity disorder*, attention or disorder, attention deficit or disorder, attention deficit-hyperactivity or disorders, attention deficit or disorders, attention deficit-hyperactivity or dysfunction, minimal brain or hyperkinetic syndrome or minimal brain dysfunction or syndromes, hyperkinetic).tw,kf. | 45,354 |
| Neurodegenerative disease/Dementia | 2 | exp *Neurodegenerative Diseases/ or Neurodegenerative Diseases.tw,kf. or(degenerative condition*, neurologic or degenerative diseases, central nervous system or degenerative diseases, nervous system or degenerative diseases, neurologic or degenerative diseases, spinal cord or degenerative neurologic disease* or degenerative neurologic disorder* or nervous system degenerative disease* or neurodegenerative disease* or neurodegenerative disorder or neurodegenerative disorders or neurologic degenerative condition* or neurologic degenerative disease* or neurologic disease*,degenerative or neurologic disorder*,degenerative).tw,kf. | 370,779 |
|  | 3 | exp *Dementia/ or Dementia.tw,kf. or (exp*Dementia, Vascular/ or Dementia, Vascular.tw,kf.) or (exp *Frontotemporal Dementia/ or Frontotemporal Dementia.tw,kf.)or (exp *Dementia, Multi-Infarct/ or Dementia, Multi-Infarct.tw,kf.) or TDP#43Proteinopath*.tw,kf. | 232,350 |
|  | 4 | exp *Parkinson Disease/ or Parkinson Disease.tw,kf. or (idiopathic parkinson# disease or lewy body parkinson# disease or paralysis agitans or parkinson# disease or parkinson#disease, idiopathic or parkinson's disease, lewy body or parkinsonism, primary or primary parkinsonism).tw,kf. | 125,047 |
|  | 5 | exp *Alzheimer Disease/ or Alzheimer Disease.tw,kf. or (exp *Tauopathies/ or Tauopath*.tw,kf.) or (acute confusional seniledementia or alzheimer dementia* or alzheimer# disease* or alzheimer disease, early onset or "alzheimer disease, familial (fad)" or alzheimer disease, late onset or Alzheimer sclerosis or alzheimer syndrome or Alzheimer type dementia or "alzheimer#type#dementia(atd)" or alzheimer type senile dementia or alzheimer's disease, focal onset or dementia, alzheimer or dementia, alzheimer type or dementia, presenile or dementia, primary senile degenerative or dementia, senile or early onset alzheimer disease or "familial alzheimer disease* (fad)" or focal onset alzheimer's disease or late onset alzheimer disease or presenile alzheimer dementia or presenile dementia or primary senile degenerative dementia or sclerosis, alzheimer or senile dementia or senile dementia, acute confusional or senile dementia, alzheimer type).tw,kf. | 187,854 |
|  | 6 | exp *Synucleinopathies/ or Synucleinopathies.tw,kf. or (synucleinopath* or a-synucleinopath* or alpha synuclein patholog*or alpha#synucleinopath* or Lewy Body Disease or Multiple System Atrophy).tw,kf. | 78,119 |
|  | 7 | exp *Cognitive Dysfunction/ or Cognitive Dysfunction.tw,kf. or (cognitive decline* or cognitive dysfunction* or cognitive impairment*OR cognitive impairment*, mild or decline*, cognitive or deterioration*, mental or disorder*, mild neurocognitive or dysfunction*,cognitive or impairment*, cognitive or impairment*, mild cognitive or mental deterioration* or mild cognitive impairment* or mild neurocognitive disorder* or neurocognitive disorder*, mild).tw,kf. | 84,618 |
| Studies | 8 | exp *Prevalence/ or Prevalence.tw,kf. or prevalence case control stud*.tw,kf. or (exp*Incidence/ or Incidence.tw,kf.) or (exp *Risk/ or Risk*.tw,kf.) or (exp *Case-Control Studies/ or Case-Control Stud*.tw,kf.) or (exp Cohort Studies/ or Cohort Stud*.tw,kf.) | 5,634,690 |
|  | 9 | 2 or 3 or 4 or 5 or 6 or 7 | 597,455 |
|  | 10 | 1 and 8 and 9 | 611 |
|  | 11 | Limit 10 to humans | 554 |

1. *PsycInfo (Ovid) (August 30, 2022)*

APA PsycInfo <1806 to August Week 5 2022> <1806 to May Week 2 2023>

| **Concept** | **Number** | **Keywords and MeSH** | **Results** |
| --- | --- | --- | --- |
| Attention-deficit/hyperactivity disorder | 1 | exp *Attention Deficit Disorder with Hyperactivity/ or Attention Deficit Disorder with Hyperactivity.tw,hw. or (addh or adhd or attention deficit disorder* or attention deficit disorder* with hyperactivity or attention deficit hyperactivity disorder* or attention deficit-hyperactivity disorder* or brain dysfunction, minimal or deficit disorder*, attention or deficit-hyperactivity disorder*, attention or disorder, attention deficit or disorder, attention deficit-hyperactivity or disorders, attention deficit or disorders, attention deficit-hyperactivity or dysfunction, minimal brain or hyperkinetic syndrome or minimal brain dysfunction or syndromes, hyperkinetic).tw,hw. | 42,500 |
| Neurodegenerative disease/Dementia | 2 | exp *Neurodegenerative Diseases/ or Neurodegenerative Diseases.tw,hw. or(degenerative condition*, neurologic or degenerative diseases, central nervous system or degenerative diseases, nervous system or degenerative diseases, neurologic or degenerative diseases, spinal cord or degenerative neurologic disease* or degenerative neurologic disorder* or nervous system degenerative disease* or neurodegenerative disease* or neurodegenerative disorder or neurodegenerative disorders or neurologic degenerative condition* or neurologic degenerative disease* or neurologic disease*,degenerative or neurologic disorder*,degenerative).tw,hw. | 30,374 |
|  | 3 | exp *Senile Dementia/ or Senile Dementia.tw,hw.or (exp *Semantic Dementia/ or Semantic Dementia.tw,hw.) or (exp *Dementia/ or Dementia.tw,hw.) or (exp *"Dementia (Senile)"/ or "Dementia (Senile)".tw,hw.) or (exp *"Dementia(Multi Infarct)"/ or "Dementia (MultiInfarct)".tw,hw.) or (exp *Multi Infarct Dementia/or Multi Infarct Dementia.tw,hw.) or (exp*"Dementia of Alzheimers Type"/ or "Dementia of Alzheimers Type".tw,hw.) or (exp*Frontotemporal Dementia/ or Frontotemporal Dementia.tw,hw.) or (exp *Dementia Paralytica/or Dementia Paralytica.tw,hw.) or (exp*"Dementia (Presenile)"/ or "Dementia(Presenile)".tw,hw.) or (exp *Dementia Praecox/or Dementia Praecox.tw,hw.) or (exp *Dementia with Lewy Bodies/ or Dementia with Lewy Bodies.tw,hw.) or (exp *Presenile Dementia/ or Presenile Dementia.tw,hw.) | 167,287 |
|  | 4 | exp *Parkinson Disease/ or Parkinson Disease.tw,hw. or (idiopathic parkinson# disease or lewy body parkinson# disease or paralysis agitans or parkinson# disease or parkinson#disease, idiopathic or parkinson's disease, lewy body or parkinsonism, primary or primary parkinsonism).tw,hw. | 37,575 |
|  | 5 | exp *Alzheimers Disease/ or Alzheimers Disease.tw,hw. or (exp *Alzheimer Disease/ or Alzheimer Disease.tw,hw.) or (exp *Alzheimer's Disease/ or Alzheimer's Disease.tw,hw.) | 69,957 |
|  | 6 | (Tauopath* or synucleinopath* or a-synucleinopath* or alpha synuclein patholog*or alpha#synucleinopath* or Lewy Body Disease or Multiple System Atrophy).tw,hw. | 4,389 |
|  | 7 | exp *Cognitive Dysfunction/ or Cognitive Dysfunction.tw,hw. or (cognitive decline* or cognitive dysfunction* or cognitive impairment*OR cognitive impairment*, mild or decline*, cognitive or deterioration*, mental or disorder*, mild neurocognitive or dysfunction*,cognitive or impairment*, cognitive or impairment*, mild cognitive or mental deterioration* or mild cognitive impairment* or mild neurocognitive disorder* or neurocognitive disorder*, mild).tw,hw. | 56,027 |
| Studies | 8 | exp *Prevalence/ or Prevalence.tw,hw. or prevalence case control stud*.tw,hw. or (exp*Incidence/ or Incidence.tw,hw.) or (exp *Risk/ or Risk*.tw,hw.) or (exp *Case-Control Studies/ or Case-Control Stud*.tw,hw.) or (exp Cohort Studies/ or Cohort Stud*.tw,hw.) | 541,884 |
|  | 9 | 2 or 3 or 4 or 5 or 6 or 7 | 272,114 |
|  | 10 | 1 and 8 and 9 | 324 |

1. *Scopus (May 17, 2023)*

| **Concepts** | **Search terms** | **Boolean** | **Results** |
| --- | --- | --- | --- |
| Attention-deficit/hyperactivity disorder | “Attention Deficit Disorder* with Hyperactivit*” OR  “addh” OR  “adhd” OR  “Attention deficit disorder*” OR  “Attention deficit hyperactivity disorder*” OR  “Hyperkinetic Syndrome” OR  “Attention Deficit?Hyperactivity Disorder*” | AND |  |
| Neurodegenerative disease | “Neurodegenerative *” OR  “Degenerative*” AND (“neurologic” OR “central nervous system” OR “spinal cord”) OR  “Dementia*”AND (“vascular” OR “multi?infarct” OR “frontotemporal”) OR  “Parkinson*” AND (“Lewy body” OR “idiopathic”) OR  “Alzheimer*” | AND |  |
| Studies | “Prevalence” OR  “Prevalence case control stud*” OR  “Incidence” OR  “Risk*” OR  “Case Control Stud*” OR  “Cohort Stud*” | AND |  |
|  | ( LIMIT-TO ( EXACTKEYWORD,"Human" ) OR LIMIT-TO ( EXACTKEYWORD,"Humans" ) ) AND ( EXCLUDE ( DOCTYPE,"re" ) |  | 928 |

*4) Web of Science (May 17, 2023)*

| **Concepts** | **Search terms** | **Boolean** | **Results** |
| --- | --- | --- | --- |
| Attention-deficit/hyperactivity disorder | “Attention Deficit Disorder* Hyperactivit*” OR  “addh” OR  “adhd” OR  ” Hyperkinetic syndrome” | AND |  |
| Neurodegenerative disease | “Neurodegenerative* disease*” OR  “Degenerative* SAME neurologic” OR  “dementia*” OR  “vascular” OR “multi?farct” OR “Synucleinopath*” OR  “Lewy Body Disease*” OR  "Multiple System Atrophy" OR  "Parkinson Disease" OR  "Parkinsonian Disorders" OR  "Basal Ganglia Disease*" | AND |  |
| Studies | “Prevalence” OR  “Prevalence case control stud*” OR  “Incidence” OR  “Risk*” OR  “Case Control Stud*” OR  “Cohort Stud*” |  | 112 |

*5) Google Scholar (May 17, 2023)*

| **Concepts** | **Search terms** | **Boolean** | **Results** |
| --- | --- | --- | --- |
| Attention-deficit/hyperactivity disorder | “Attention Deficit Disorder* Hyperactivit*” OR  “addh” OR  “adhd” OR  ” Hyperkinetic syndrome” | AND |  |
| Neurodegenerative disease | “Neurodegenerative* disease*” OR  “Degenerative* SAME neurologic” OR  “dementia*” OR  “vascular” OR “multi?farct” OR “Synucleinopath*” OR  “Lewy Body Disease*” OR  "Multiple System Atrophy" OR  "Parkinson Disease" OR  "Parkinsonian Disorders" OR  "Basal Ganglia Disease*" | AND |  |
| Studies | “Prevalence” OR  “Prevalence case control stud*” OR  “Incidence” OR  “Risk*” OR  “Case Control Stud*” OR  “Cohort Stud*” | AND | 255 |

# Supplementary Table

Supplementary Table 1. Detailed information about each study included in the review.

| **Study** | **Aim** | **Start Date** | **End Date** | **Database** | **Inclusion Criteria** | **Exclusion Criteria** |
| --- | --- | --- | --- | --- | --- | --- |
| Curtin, 2018 (1) | To determine if ADHD or dopaminergic treatments alter the risk of diseases of the basal ganglia and cerebellum, including Parkinson’s disease | 01-Jan-96 | 31-Dec-16 | Utah Population Database | (1) born between 1950-1992, (2) at least 20 years of age on 31 December 2011 or date of last follow-up, if earlier and (3) resident of Utah on or after 1 January 1996 (baseline) as determined by vital records and other demographic data in the Utah Population Database | (1) diagnosis of HIV, (2) any history of abuse of amphetamines or methamphetamines, other illicit drugs, or alcohol abuse based on an ICD-9 diagnosis in the electronic medical records, and (3) alcohol abuse disorders |
| Du Reitz, 2021 (2) | To investigate the phenotypic and aetiological associations between ADHD and a wide range of physical health conditions | 01-Jan-05 | Not reported | Swedish National Patient Register | Full sibling and maternal half-sibling pairs born between Jan 1, 1932, and Dec 31, 1995 | Individuals who died or emigrated before Jan 1, 2005, excluded twins. |
| Dobrosavljevic, 2022 (3) | To investigate the association of ADHD with dementia and MCI, as well as accounting for comorbid conditions, education, head injuries, other developmental disorders, and sex | 01-Jan-01 | 31-Dec-13 | Swedish registers (Total population Register, National Patient Register, Cause of Death Register) | Individuals born between 1932 and 1963, who were alive and resided in Sweden in 2001 | (1) Individuals who emigrated from Sweden and died before 2001, and before age 50, and (2) those who immigrated to Sweden after 2001 and aged 50 and above |
| Fan, 2020 (4) | To determine whether PD patients exhibited a greater propensity for the prior diagnosis of ADHD than a control group of matched patients without PD | 01-Jan-00 | 31-Dec-13 | Taiwan’s Longitudinal Health Insurance Database | PD patients had to have at least three or more outpatient visits or hospital admissions AND received one PD medication | unknown/none |
| Fluegge, 2018 (5) | To ascertain whether a ten-year lagged measure of ADHD predicts hospitalization for two dementia subtypes, LBD and AD | Not reported | Not reported | United States Healthcare Cost and Utilization Project | unknown | unknown |
| Golimstok, 2011 (6) | To test the hypothesis that ADHD symptoms may precede DLB onset | 2000 | 2005 | Argentina’s Italian Hospital Medical Care Program | Patients with dementia who met NINCDS-ADRDA or DLB consensus criteria and MMSE 14-26 or CDR 1-2. Controls were selected from the same general practice list | Formal examination showed evidence of any other brain disorder or physical and or mental illness sufficient to contribute considerably to the clinical picture |
| Tzeng, 2019 (7) | To investigate the association between adults with ADHD and the risk of developing dementia | 01-Jan-00 | 31-Dec-10 | Taiwanese Longitudinal Health Insurance Database | Three ADHD outpatient visits within 1 year, OR one ADHD inpatient visit | (1) ADHD before 2000, (2) drug dependence and nondependent abuse of drugs, (3) dementia before tracking, (4) gender unknown, (5) age <18 |

Notes. AD, Alzheimer’s Disease; ADHD, Attention-Deficit Hyperactivity Disorder; CDR, Clinical Dementia Rating Scale; DLB, Dementia with Lewy Bodies; MCI, Mild Cognitive Impairment; MMSE, Mini Mental State Examination; NINCDS-ADRDA, National Institute of Neurological and Communicative Diseases and Stroke/Alzheimer's Disease and Related Disorders Association; HIV, Human Immunodeficiency virus; ICD, International Classification of Diseases; LBD, Lewy Body Diseases; PD, Parkinson’s Disease

# References

1. Curtin K, Fleckenstein AE, Keeshin BR, Yurgelun-Todd DA, Renshaw PF, Smith KR, et al. Increased Risk of Diseases of the Basal Ganglia and Cerebellum in Patients with a History of Attention-Deficit/Hyperactivity Disorder. *Neuropsychopharmacology* (2018) 43(13):2548-55. Epub 20180912. doi: 10.1038/s41386-018-0207-5.

2. Du Rietz E, Brikell I, Butwicka A, Leone M, Chang Z, Cortese S, et al. Mapping Phenotypic and Aetiological Associations between Adhd and Physical Conditions in Adulthood in Sweden: A Genetically Informed Register Study. *Lancet Psychiatry* (2021) 8(9):774-83. Epub 20210706. doi: 10.1016/S2215-0366(21)00171-1.

3. Dobrosavljevic M, Zhang L, Garcia-Argibay M, Du Rietz E, Andershed H, Chang Z, et al. Attention-Deficit/Hyperactivity Disorder as a Risk Factor for Dementia and Mild Cognitive Impairment: A Population-Based Register Study. *Eur Psychiatry* (2021) 65(1):1-19. Epub 20211220. doi: 10.1192/j.eurpsy.2021.2261.

4. Fan HC, Chang YK, Tsai JD, Chiang KL, Shih JH, Yeh KY, et al. The Association between Parkinson's Disease and Attention-Deficit Hyperactivity Disorder. *Cell Transplant* (2020) 29:963689720947416. doi: 10.1177/0963689720947416.

5. Fluegge K, Fluegge K. Antecedent Adhd, Dementia, and Metabolic Dysregulation: A U.S. Based Cohort Analysis. *Neurochem Int* (2018) 112:255-8. Epub 20170812. doi: 10.1016/j.neuint.2017.08.005.

6. Golimstok A, Rojas JI, Romano M, Zurru MC, Doctorovich D, Cristiano E. Previous Adult Attention-Deficit and Hyperactivity Disorder Symptoms and Risk of Dementia with Lewy Bodies: A Case-Control Study. *Eur J Neurol* (2011) 18(1):78-84. Epub 2010/05/25. doi: 10.1111/j.1468-1331.2010.03064.x.

7. Tzeng NS, Chung CH, Lin FH, Yeh CB, Huang SY, Lu RB, et al. Risk of Dementia in Adults with Adhd: A Nationwide, Population-Based Cohort Study in Taiwan. *J Atten Disord* (2019) 23(9):995-1006. Epub 20170619. doi: 10.1177/1087054717714057.
